# “*Once you get one maternal death, it's like the whole world is dropping on you*”: experiences of managing maternal mortality amongst obstetric care providers in Ghana

**DOI:** 10.1186/s12884-022-04535-z

**Published:** 2022-03-14

**Authors:** Anna Stabnick, Michael Yeboah, Johnny Arthur-Komeh, Frank Ankobea, Cheryl A. Moyer, Emma R. Lawrence

**Affiliations:** 1 School of Public Health, University of Michigan, 1415 Washington Heights, Ann Arbor, MI 48109 USA; 2 Directorate of Obstetrics and Gynaecology, Komfo Anokye Teaching Hospital, Okomfo Anokye Road, Kumasi, Ghana; 3 Department of Obstetrics and Gynecology, KNUST-SMD, Kumasi, Ghana; 4 Global REACH, Department of Obstetrics & Gynecology, University of Michigan Medical School, 1500 E. Medical Center Dr, Ann Arbor, MI 48109 USA

**Keywords:** Impact of maternal mortality, Experiences with maternal mortality, Support for obstetric providers, Maternal mortality in LMIC

## Abstract

**Background:**

Maternal mortality has a significant global impact, especially in low-resource settings. Little prior research has been conducted on the potential effects of poor maternal outcomes on the personal and professional well-being of healthcare providers. This study explores the in-depth experiences and perspectives of obstetric providers in Ghana who work in a setting with frequent maternal mortalities.

**Methods:**

This is a qualitative study of semi-structured interviews conducted at the Komfo Anokye Teaching Hospital in Ghana. Participants were obstetric healthcare providers, defined as midwives, house officers currently rotating on the obstetrics/gynecology service, and obstetrician/gynecologists at any training or practice level (residents, fellows, and specialists). Interviews were audio-recorded, transcribed verbatim, and uploaded into NVivo for qualitative analysis. Using the Attride-Stirling qualitative model, an incremental and iterative process was used to code interviews with keyword phrases and develop a framework of organizing and global themes.

**Results:**

Interviews were conducted with 27 participants—15 midwives and 12 physicians (three obstetrician/gynecologist residents, six obstetrician/gynecologist specialists, and three house officers), with sample size determined by data saturation. Obstetric providers’ experiences in a setting with frequent maternal mortalities were dependent on their level of preparedness to manage maternal mortalities and the workplace environment. Providers’ level of preparedness was dependent on both the training they had received on the medical management of obstetric emergencies, as well as a lack of training on the mental health aspects of coping with maternal mortality. The impact of the workplace environment was dependent on systems failures and limited resources, blame from colleagues and supervisors, and a lack of support in the workplace. In turn, obstetric providers’ experiences managing frequent maternal mortalities impacted their clinical care performance and mental health.

**Conclusions:**

Maternal deaths have profound personal and professional impacts on the healthcare providers who manage them. A large need exists for additional institutional training and support for obstetric providers who manage maternal mortality, especially in low-resource settings like Ghana.

## Background

Maternal mortality has a significant global impact on patients, their communities, and the obstetric providers who care for them [1]. Every day, more than 800 people die from preventable complications of pregnancy [2]. Worldwide, rates of maternal mortality are decreasing, with the World Health Organization estimating a 38% drop in maternal deaths between 2000 and 2017 [2]. However, there are profound disparities in maternal outcomes by race, socioeconomic status, and geographic location [3, 4]. Rates of significant maternal morbidity and maternal mortality remain unacceptably high in low- and middle-income countries (LMICs), where 94% of all maternal deaths occur [1, 2]. Rates of maternal mortality are highest in sub-Saharan Africa, with a maternal mortality ratio of 533 maternal deaths per 100,000 live births [5].

Most maternal deaths are caused by preventable and treatable complications, including postpartum hemorrhage, infection, hypertensive disorders of pregnancy, and unsafe abortion [6]. Importantly, births attended by skilled healthcare providers have better outcomes [7]. However, the availability of skilled obstetric providers, including midwives, nurses, general physicians, and especially obstetricians, is limited in sub-Saharan Africa [8, 9]. In Ghana, despite a highly trained workforce of obstetric providers in urban centers, [10, 11] high maternal mortality rates persist due to limited resources, systems failures, and socioeconomic factors [12, 13].

The management of maternal mortality is an intense experience for healthcare providers, with potential impacts on mental health [14] and physical health [15]. In addition, management of maternal death may result in burnout and workplace distress [16]. Despite the disproportionate burden of maternal deaths in low-resource settings, little research has been conducted to explore the effects that poor maternal outcomes can have on the personal and professional well-being of obstetric providers in low-resource settings like Ghana.

Prior quantitative research among obstetric providers in Ghana identified that managing maternal mortalities has a large negative impact on providers, with the majority reporting inadequate support, large emotional tolls, and feelings of guilt or shame [17]. However, gaps exist in understanding how personal, professional, and workplace factors impact providers’ experiences of maternal mortality. This study uses a qualitative interview design to further explore the in-depth experiences and perspectives of obstetric providers in a setting with frequent maternal mortalities.

## Methods

### Design

Our study used grounded theory to explore obstetric providers’ experience managing maternal mortality at the Komfo Anokye Teaching Hospital (KATH) in Kumasi, Ghana. An explanatory qualitative design was selected to build upon findings from a quantitative analysis of survey questions completed by 270 obstetric providers at KATH [17]. Grounded theory is a qualitative approach for collecting and analyzing data without imposing previously constructed theoretical frameworks [18, 19]. This approach was used to capture participants’ perspectives without assuming they would conform to the researchers’ ideas about managing maternal mortality in Ghana.

### Study site

This study was conducted at KATH, which is Ghana’s second largest teaching hospital and is a tertiary referral hospital for patients throughout central Ghana. KATH manages approximately 10,000 deliveries each year. The OBGYN department is staffed by OBGYN specialists, OBGYN resident trainees, midwives, and house officers. House officers spend six months of their two-year training period as general physicians rotating in the OBGYN department. The OBGYN Department has formal, well-established programs to train OBGYN residents, house officers, and midwife students.

### Participants

At KATH, the management of pregnant women is conducted by three groups of healthcare providers: 1) certified nurse midwives (“midwives”), 2) doctors with a specialization in obstetrics and gynecology (“OBGYNs”), and 3) doctors who recently graduated from medical school and are rotating through core clinical services to gain additional training (“house officers”). Participants in this study were obstetric providers whose primary clinical appointment was at KATH. Inclusion criteria were an occupation as a clinical obstetric provider, participation in the initial survey study, and experience with maternal mortality [17]. Obstetric providers were defined as midwives, house officers currently rotating with the OBGYN department, or OBGYNs at any level of training or practice (residents, fellows, specialists, and consultants). Experience with maternal mortality was defined as direct personal management of a maternal death, working as part of a clinical team who managed a maternal death, or having workplace interactions with colleagues impacted by maternal death. Exclusion criteria were a non-obstetric healthcare provider, and providers whose primary clinical site was not KATH.

### Recruitment

Purposive sampling was used to select a range of obstetric providers who had meaningful experiences with maternal mortality. All 270 participants in the initial quantitative study [17] were asked if they were interested in participating in a follow-up interview and 104 responded “yes” and provided their WhatsApp contact number for research followup. From those, potential participants were stratified by role (OBGYNs, house officers, and midwives) to ensure a diversity of views. Participants were sampled from each group, in proportion to the size of the group. Sampling and interviews were continued until data saturation was achieved [20], which was determined by an ongoing review of interview content to decide when new interview data was redundant of already collected data [21]. In total, 61 obstetric providers were contacted to schedule an interview. Fifteen providers were no longer working at KATH, leaving 46 eligible participants; 17 did not respond to attempts to schedule an interview and two declined to participate. Interviews were conducted with 27 total participants, which included 15 midwives and 12 physicians (three OBGYN residents, six OBGYN specialists, and three house officers). Qualitative trustworthiness was established based on the credibility of the participants as obstetric providers. All participants had experience with maternal mortality, and the vast majority of participants (all OBGYNs, all house officers, and 12 of 15 midwives) had direct personal experience managing a maternal death. Of the three midwives without direct personal experience, all had managed women who subsequently died during later shifts, or were part of a team that was managed a maternal death. Quotes from these three midwives are specifically denoted in the results section.

### Procedures

Due to the ongoing COVID-19 pandemic, a virtual WhatsApp platform was used to contact participants and perform interviews. WhatsApp is a commonly used, no-cost, electronic communication platform, which includes a video call feature. Interviews were conducted by an American female public health student. She received training on qualitative interviewing techniques [22] and orientation to the sensitive nature of interview topics and culturally appropriate approaches to asking about maternal mortality. All interviews were performed in English, which is an official language in Ghana used for medical education. Electronic informed consent was obtained from all participants. As part of the consent process, participants were informed they could skip any question that caused significant distress and were able to pause or stop the interview at any time. If mental health support was requested, contact information was provided for a psychiatrist at KATH who was aware of this research. A flash drive valued at $4 USD was provided as an incentive to participate.

Semi-structured interviews were conducted from July 2020 to November 2020, consisting of five sections, and lasting approximately 40 min. Each section contained open-ended introductory questions, as well as a series of more specific prompts. Non-verbal communication was not formally assessed, however was utilized by the interviewer to guide followup probes. Section 1 asked about the study participants’ experiences with maternal mortality, including feelings of control over patient outcomes. Section 2 focused on preparedness to handle maternal mortality. Section 3 investigated sources of support and the adequacy of this support. Section 4 asked about the work environment and attitudes of colleagues. Section 5 focused on the emotional and psychological impacts of maternal death on providers.

### Analysis

Interviews were audio-recorded, transcribed verbatim, and uploaded into NVivo 12 (QSR International, Burlington, MA USA) for qualitative analysis. Using an incremental and iterative process, two researchers (A.S. and E.R.L) created a codebook consisting of 21 keyword phrases. After the list achieved stability, the final coding process was repeated for all transcripts. A comment could be coded with multiple keyword-phrases, to represent multi-themed ideas. Using the Attride-Stirling model of qualitative analysis [21], a framework of organizing and global themes was developed.

## Results

**Fig. 1:**
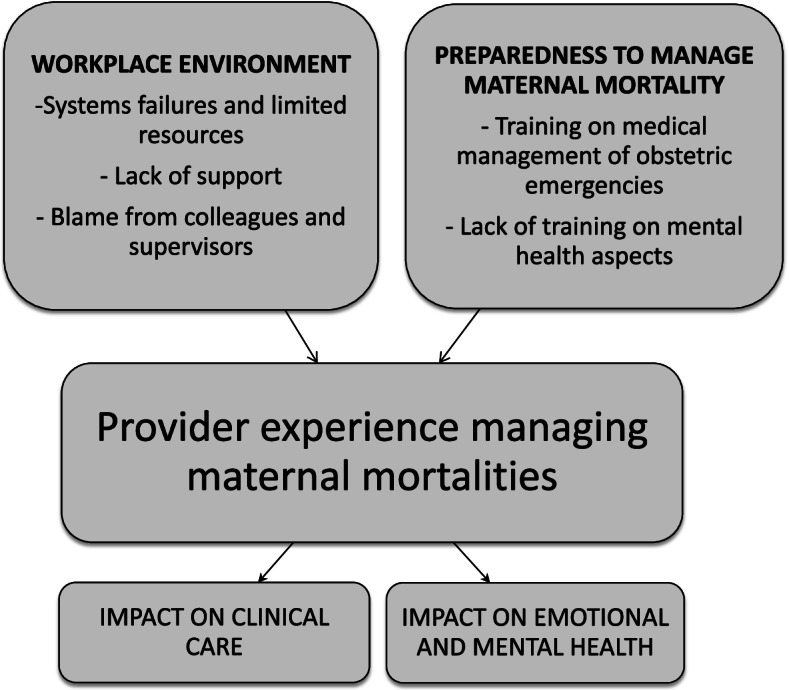
Framework for provider experiences with maternal mortalities

Interviews indicated that obstetric providers’ experiences in a setting with frequent maternal mortalities were primarily dependent on their level of preparedness to manage maternal mortality, and the workplace environment. Preparedness was dependent on both the training they had received on the medical management of obstetric emergencies, as well as lack of training on the mental health aspects of coping with maternal mortality. The impact of the workplace environment was dependent on systems failures and limited resources, blame from colleagues and supervisors, and a lack of support in the workplace. In turn, experiences managing frequent maternal mortalities impacted the clinical care performance and emotional and mental health of obstetric providers (Fig. 1).

### Preparedness to manage maternal mortality

***Training on the medical management of obstetric emergencies ***was identified as an important, positive factor. When staff received training related to maternal mortalities, it was almost always focused on the procedural aspects of their practice, the auditing process, or breaking bad news to family members. These components of training helped hospital staff to feel more prepared to handle maternal mortalities, but many expressed that they would benefit from additional training.

*Oh, we have training on postpartum hemorrhage...when a woman comes to deliver and the woman is bleeding. What we should do with the woman in the ward in order to save the life...And I am grateful for that training. And so I think that we will know how to handle them well. (Midwife, 3rd year)*^1^

Other things that helped providers feel more prepared with the medical side of maternal mortality management were experiences with past maternal mortalities, feeling like their colleagues were well-prepared, and feeling motivated or driven in their work to continue learning and doing better.

*With the training, I have acquired some skills and some composure that when cases come in, you don't panic. And luckily for me... I usually have senior colleagues around to help out... So, yes, the junior doctors have some control over situations when it comes to obstetrics management. (House officer, 1*^*st*^* year)*

Most providers said that while they felt prepared to handle maternal mortalities, they were aware there were some cases they knew they would not be able to handle well or did not have control over.

Some [maternal deaths], there is nothing that you can do about it. It's inevitable and it's bound to happen and it happens. (Midwife, 12^th^ year; has not directly managed a maternal death)

Despite receiving training on the medical management of obstetric emergencies, most providers reported a ***lack of preparedness and training on mental health aspects*** of managing maternal mortalities. They had never received training or guidance on processing poor outcomes or managing workplace stress. Providers emphasized how important it was for those working in obstetrics in Ghana to have good coping mechanisms, but many did not have those skills.

*I've not been given any formal training on how to handle stress, when we have bad outcomes and how to handle them... These things come from my own personal experiences...It's important that we are able to gain training and education on how to deal with certain things like that. Because it will put us in a better state of mind and a better place to be able to handle such issues. (OBGYN resident, 3rd year)*

Providers felt that the most helpful things for them to be prepared to handle deaths mentally and emotionally were past experiences and guidance from supervisors or superiors. Almost all participants felt that training in this area would be very helpful, as well as more facilitated conversations among colleagues to break the culture of silence that many of them felt around this issue. Many providers felt that there was no way that they could be fully prepared for handling maternal mortalities, but that having training would help them know what to expect and to have more tools and strategies.

*I think that it should be something that should be part of...the orientation or as part of continuous professional development. These things can be added [on] how to deal with maternal mortality. Because we never, we never talk about these things. It’s something that I think we can do... It would be a big topic to have psychologists come in to speak to us. (OBGYN specialist, 11th year)*

### Workplace environment impacting experience of managing maternal mortality

In the workplace, ***system failures and limited resources*** were reported by providers as the greatest challenge they faced in managing maternal mortalities. These systems issues led to a loss of a sense of control over critically ill patient situations. Many commented that deaths often would be preventable if certain system failures had not taken place.

*We [had a] lot of cases where you would feel you could save the patient, but due to a lot of factors, you would realize that the patient ends up passing away when you did not expect it...I do blame the system, the health system itself (OBGYN resident, 3*^*rd*^* year)*

*I think our resources...in the facilities aren't that good...because as one midwife, you can attend to about sixty [patients]... and by the time you finish with one [patient] you are just exhausted so you can't go into proper counseling. (Midwife, 10th year)*

Some of the most commonly reported system challenges discussed by providers were a lack of availability of blood products, a high patient volume resulting in an overload of work, and poor communication from referral centers.

*Once she reached here, she was so pale, she was so pale... This woman had a very good surgery, but there wasn't adequate blood. So at the end of the day, the woman died... We had run out of the blood that was necessary for her… And then, after a few hours, a similar case came around...So guess what I did. I had to go out of the teaching hospital to another hospital, send for blood myself. And I brought it to the hospital and we were able to manage that woman. And as I speak right now, she made it, but she's in the ICU today. I checked on her and she's getting better. And I feel so bad about the one that died because I think the only thing we needed at that point was blood. (OBGYN resident, 3*^*rd*^* year)*

Across all roles, participants reported an overall ***lack of support in the workplace.*** Things that participants reported as being specifically unhelpful or counterproductive were silence around the issue and acting as though maternal death was a normal occurrence.

*I haven't seen a single time that there has been a maternal mortality and someone says, ‘oh, this doctor, after managing the case, is very emotional, and very sad. Let me just go and talk to him’…I feel like there should be some contribution from the senior colleagues…or the members of the team. But it doesn't exist. If it does, I haven't experienced it. (OBGYN resident, 3*^*rd*^* year)*

*More could be done, especially for the junior doctors, because most people when they...are hit with maternal mortality, we begin to have second thoughts about the profession as a whole. (House officer, 1st year)*

If workplace support did occur, it came in the form of encouragement from colleagues, and especially from supervisors; this was reported as being the most helpful. Other forms of workplace support reported included giving days off to providers to cope away from the workplace, as well as providing increased procedural support and supervision from more senior colleagues.

*[My supervisors] tell you, it's your work, you are saving even more people than the one you have lost during your past experiences, you have delivered a lot of babies, you have helped a lot of clients, who are now alive. So, I think that's what keeps me going. (Midwife, 10th year)*

Many providers expressed a desire for increased mental health support, consisting of a counseling unit or department within the hospital with mental health professionals specifically for providers to seek support. Many felt that when a death occurs, the involved providers should be referred to psychologists or other counseling professionals, but that this is currently not the case. Providers believed that accessing this support could make the difference for a struggling provider in whether or not they continued to practice. Further clarifications revealed that while there are counseling and psychiatry services in the hospital, referrals are usually made to patients and families, but rarely do providers use these services. If services were more easily accessible and tailored more towards provider support, the vast majority of participants expressed that they would use these services.

*I wish we had [mental health support] because sometimes you really need to talk to somebody, maybe after the death, then you think if you could talk to somebody...and pour it out, you could be free, you could be relieved. But there's no system in place for now, for that. So you just get by it yourself. (Midwife, 10th year)*

In addition to a lack of support in the workplace, many participants also experienced ***blame from colleagues and superiors***.

*Uh huh, you are blamed. Everybody blames you because you were [working] when a death occurred. Forgetting that there was a turn of events that resulted in the death...You are going to be blamed by colleagues, by superiors, and people look at you as if you are not competent. They tag you, that you are not competent. (Midwife, 12th year; has not directly managed a maternal death)*

Blame was expressed more consistently by junior trainees and midwives, compared to more senior OBGYNs.

*When a maternal death occurs, at times people feel they are being attacked...They feel they have been blamed for the death which occurred because of one lapse or the other. So at times people are a bit afraid even to take care of patients when they come with an obstetric emergency, especially the midwives -- she is scared because if something happens she will be blamed....[We should] address the problem and not victimize the person....So people get demoralized, and they are not motivated to take care of their next patient who comes. If they do, they are, it’s like walking on eggs. They are so scared. (OBGYN specialist, 7th year)*

Blame was felt in both informal conversations with colleagues and during formal debriefing processes. Participants described monthly maternal mortality meetings and audits, which occur shortly after each death. Monthly meetings are forums to discuss hospital and departmental operations and management changes, while audits are specific reviews of patient deaths. Participants noted that the meetings sometimes are turned into a "blame game."

*A lot of people want to avoid going [to] maternal mortality meetings because they feel that it is a platform for witch-hunting. Where you would just be prodded with a lot of questioning, a lot of rebuke, a lot of criticisms, and you would just come out and you feel that, remind me do I really need to do this? Am I ready for this? And, am I in the position to take care of any patient again? (OBGYN resident, 3rd year)*

An additional issue reported with the meetings and audits was that there tended to be a power imbalance with who was present, who got to provide input, and who received feedback. While OBGYNs were universally included in the audits, several midwives commented that they were not included and were not informed of the audit’s conclusions.

Despite the criticisms of these formal debriefs, nearly all participants commented on the helpfulness of these meetings to learn from mistakes and discuss what went wrong. Many commented that it gave them something to focus their energy on and be proactive going forward, as well as gaining a sense of closure.

*During [maternal mortality meetings], the case is presented and then we find the mistakes that were done, what could have been done better to prevent such deaths...Yes, it’s very helpful... It helps you not to make the same mistake twice. And at those meetings you have senior colleagues who have more experience than you have...they help you to know which ways are better when it comes to managing certain conditions and what the guidelines say. (House officer, 1st year)*

### Impact of maternal mortality on obstetric providers

Many participants commented that experiences with maternal mortality had an ***impact on clinical care performance*** by making them more cautious and thorough in their patient care. They discussed becoming more attentive to certain clinical signs or signals and spending more time assessing patients. Many commented that they learned from the clinical presentation and management of mortalities that they experienced, which was enhanced by audits and informal debriefs with coworkers.

*I tried to read more, I tried to find out more...We had a lot of protocols. We printed them and laminate[d] them at our delivery ward and then in our office... I spoke to colleagues. I spoke to people who had more experience than I did... I dealt with it pretty good. I learned a lot of things. And I made sure referrals [were] always on point. I didn't want to delay any other case or have any complications at hand...It did help me, yes. (Midwife, 7th year)*

*[The maternal death] really helped me to be very particular or careful of the care of other patients. When it comes to monitoring, documentation, keeping records very well or keeping an eye on the patient[s]. (Midwife, 11th year).*

Some commented that their experiences motivated them to try to fix issues in the healthcare system in Ghana to prevent future mortalities on a larger scale.

*For the [maternal deaths] that you cannot do much about…It tells us how much we should advocate for governments to put terms in place. Because as obstetricians, we are also women advocates. So we also have to advocate for empowering women, for ensuring women’s health...And not just being clinicians. (OBGYN specialist, 10th year)*

Negative impacts on patient care included reported symptoms of post-traumatic stress disorder (panic attacks, flashbacks, hesitancy to approach certain cases) and experience with mortalities slowing down and interfering with work due to their emotional impact.

*The first encounter with another delivery, I realized I was sweating all of a sudden, I was sweating and I was a bit shaky. So I had to leave and call my other colleagues to come in and then conduct the delivery. It happened for...I think five more cases or so... I think that was anxiety or some panic attack that I was having. (Midwife, 5th year)*

In addition to impacts on clinical care, providers cited a significant ***impact on emotional and mental health.*** Providers uniformly reported feeling bad or sad after experiencing maternal deaths. Many expressed concern for the wellbeing of the mother's child or family that was left behind after the death.

*It makes me very, very, very sad and also the memories of maternal death stays with me for a very long time...And, there have been times I will also be thinking about what's going to happen to the family of this woman who died. How are the kids going to cope and all that. (OBGYN resident, 3rd year)*

Providers expressed that the emotional impact was greater when the provider had a closer connection to the patient or had cared for them longer, or if the death was either unexpected or not fully understood. Words used to describe the emotional impact included “traumatizing”, “stressful”, “sad”, “bad,” “difficult”, “worrisome”, “hurtful”, “ashamed”, “guilty”, “painful”, “devastating”, “terrible”, “draining”, “disheartening”.

Once you get one maternal death, it's like the whole world is dropping on you. (Midwife, 10th year)

Many also commented that there are deaths that they have never been able to fully cope with and still carry with them, often going on to recount the full experience during their interviews. Many participants shared that the emotional impact of deaths impacted their practice.

I look fine on the outside. Nobody even knew I hadn't slept for 3 nights. I looked okay but within me, I knew what it was eating me up. (Midwife, 7th year)

In addition to experiencing anxiety and panic symptoms at the workplace, more pervasive anxiety was also common among providers. A few providers commented that these mental health issues were rarer and only occur in "extreme" cases. Mild cases of depression were also self-reported. Common symptoms reported included having flashbacks, shivering or trembling or other physical signs of panic attacks, and changes in sleeping patterns.

*Oh, it's terrible, honestly, I become distraught. I have sleeplessness, I have insomnia. I have loss of appetite. I cry a lot. And I have a lot of regrets, and I think I have minor, just minor depression, when I lose a patient like that, but then since the cycle never ends, I think I tend to get over it quickly. (Midwife, 8th year)*

## Discussion

Among a range of obstetric providers in Ghana, this study demonstrates the profound impact of managing maternal mortalities on the personal and professional lives of providers. Apart from increased blame felt by junior trainees and midwives, similar themes were expressed across clinical roles, trainee levels, and years of experience.

In our study, providers’ experiences are dependent on their preparedness to manage maternal mortality, which is positively impacted by training on the medical management of obstetric emergencies, and negatively impacted by the lack of training on the mental health aspects. The literature supports the positive impact of training on obstetric emergencies, which results in increased provider confidence and comfort, enhanced knowledge and skills, and a reduction of maternal deaths in low-resource settings[23–25]. Although the cost effectiveness of obstetric emergency training in LMICs has not been conclusively demonstrated, widespread benefits likely outweigh the relatively small costs [26]. Conversely, in both low-resource and high-resource settings, minimal training is provided on the emotional aspects of dealing with maternal death and workplace stress [17, 27]. Providers typically feel under-prepared to cope with death of their patients, and desire additional formal training in this realm [16, 27].

In our study, providers’ experiences managing maternal mortality are also dependent on the workplace environment. Providers emphasize their frustrations with the systems challenges they encounter, which often result in avoidable maternal deaths. This is compounded by a lack of consistent support from colleagues and superiors, and workplace blame. In the literature, medical providers consistently report support from colleagues and supervisors as a positive factor in productive coping with traumatic workplace events, including maternal death [28]. Peer support in the workplace has been shown to improve rates of emotional exhaustion and burnout [29]. However, many studies demonstrate that institutional support is inadequate [14, 30]. The importance of workplace culture in mediating responses to workplace stress, and the tendency toward negative environments in medicine, is commonly cited in the literature [31]. Workplace cultures of the blame can result in burnout and desire to leave the medical profession [26, 31]. The concept of blame following a death in the workplace is complex. Formal debriefing processes, audits, and departmental “morbidity and mortality” meetings are essential for learning and quality improvement, and many providers view them as educational and therapeutic [32]. However, discussions about poor outcomes can result in an increased emotional response, from both the providers who cared for the patient who died, as well as colleagues and supervisors, and lead to real or perceived blame [33].

Finally, our study demonstrates that experiences with maternal death are transformative experiences for many providers, with significant impacts on clinical care performance and emotional and mental health. Consistent with the literature, impact on clinical care performance is complex, and can be both negative and positive. In many cases, experiences with maternal mortality leads to workplace anxiety and trauma, and a decreased ability to concentrate on subsequent patient encounters [34–36]. Despite these consequences, positive impacts include a renewed dedication to personal, departmental, and systems improvement following poor outcomes [36, 37]. Universally, management of patient deaths results in profound negative emotional impacts on healthcare providers [36, 37]. When focusing specifically on maternal mortality, studies done in high-income countries demonstrate that maternal death can result in strong emotional responses like grief, guilt, and shame, [27] as well as mental health issues, including depression and anxiety [30]. Although research in LMICs like Ghana is limited, studies on the impact of maternal death[14] and stillbirth[38] on Ghanaian midwives also demonstrate emotional distress and self-blame.

### Strengths and limitations

Strengths of this study include a qualitative design, which is well-suited for investigating the intense and nuanced experiences of providers. The sample size is appropriate for qualitative research and our conclusions are strengthened by foundational quantitative research in the same population [17]. Due to the COVID-19 pandemic, the original plan for in-person interviews was replaced with a virtual design using video calls. Virtual interviews may lose some of the rapport-building and nuanced nonverbal cues that are present with in-person interactions [39]. However, video calls do offer participants the advantages of scheduling ease and the ability to choose the location of their interview that they feel is most comfortable and private. Our study was conducted at a single tertiary hospital setting in Ghana, and thus themes that emerged from interviews may be unique to this setting. This study site was selected due to it being a large referral center with a high burden of maternal mortalities, as well as a teaching hospital with multiple levels and types of obstetric trainees. This included three providers who had not personally managed a maternal mortality but were able to share their perspectives on their training and preparedness, as well as experiences with colleagues who managed maternal mortality. As we intentionally sampled from multiple types of obstetric providers, we believe this study demonstrates findings from diverse clinical roles and represents a depth and breadth of experience. Two of the study authors work in the same hospital as the study site. To limit bias and increase the comfort of participants to share their perspectives fully, interviews were conducted by a researcher who was external to the study site. The study team was diverse, consisting of an American OBGYN, a public health faculty and public health student, as well as two Ghanaian OBGYNs.

### Policy and practice implications

Hospital and departmental leadership should develop organizational approaches to empowering staff to cope with maternal death [27]. Approaches include training during medical/midwifery school and professional development activities to help prepare providers for the emotional aspects of maternal death. After deaths occur, debriefing sessions are a systematic approach aimed at providing emotional support, education about normal and maladaptive coping and stress responses, and tools for providers to self-identify warning signs [40]. Structured bereavement debriefing sessions, focused on providing emotional support and building skills to manage grief, were found to be helpful by healthcare providers managing pediatric deaths [28, 41], and could be applied to the obstetric setting. In addition, formal counseling and psychiatric services could be offered to obstetric providers who manage maternal death, either as a broad opt-out occupational health policy or in specific cases.

## Conclusions

Overall, it is critical to recognize that maternal deaths can have important personal and professional impacts on the healthcare providers who manage them. In LMICs, obstetric providers are challenged to work in settings with frequent maternal mortalities amidst the other stressors of providing care in low-resource settings. A large need exists for additional institutional training and support for providers who manage maternal mortalities, especially in LMICs.

## Data Availability

The datasets used and analyzed during the current study are available from the corresponding author on reasonable request.

## Abbreviations

LMICs: Low- and middle-income countries
OBGYN: Obstetrics and gynecology
KATH: Komfo Anokye Teaching Hospital

## Footnotes

1 Quotations are identified by the clinical role and the number of years the individual has been working in that role.
